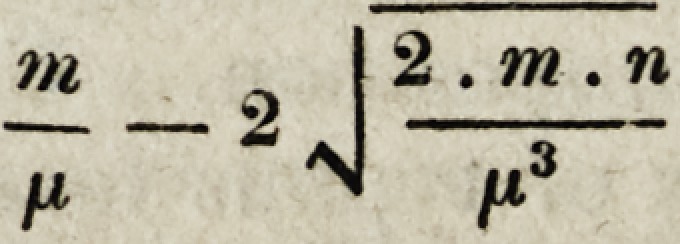# General Principles of Medical Statistics, or a Development of the Rules Which Ought to Preside over the Use of Numbers in Medical Investigations

**Published:** 1841-07

**Authors:** 


### BRITISH AND FOREIGN

### MEDICAL REVIEW,

FOR JULY, 1841.

### PART FIRST.

### Analytical and Critical Reviews.

### Art. I.

*Principes généraux de Statistique Médicale, ou Développement des Règles qui doivent présider à son emploi.* Par Jules Gavarret, Ancien Elève de l'Ecole polytechnique.—*Paris*, 1840. 8vo, pp. 312.

*General Principles of Medical Statistics, or a Development of the Rules which ought to preside over the use of Numbers in Medical Investigations.* By Jules Gavarret, formerly a pupil of the Polytechnic School.—*Paris*, 1840.

Medicine is both a science and an art, a collection of principles on the one hand, and of practical applications of those principles to individual cases on the other; as a science most imperfect, as an art most difficult. Originally medicine, like almost every other branch of human knowledge, was a mere art; that is to say, it possessed no rules, but those who practised it did in one case that which they had seen or known to be useful in an individual case which resembled it. Medicine ceased to be an art, and began to wear the form of a science, when principles took the place of facts; in other words, when men began to collect, arrange, and analyze individual and like instances, and to express that which was common to them all in the form of propositions or aphorisms, which might serve for instruction to the student and guides to the practitioner. In this sense of the term, Hippocrates was the father of physic—of physic not as an art but as a science, and those only deserve the name of successful cultivators of the science who deduce principles from the facts collected by themselves or others; he who deals merely with individual instances, whether as the groundwork of his opinions or the guides of his practice, deserves no higher praise than that awarded to the successful practitioner of an *art*.

The art of medicine, however, which preceded the science differed widely from the art which sprang from the science: the one, dealing only with individual cases, was mere empiricism; the other, establishing principles, and reapplying those principles to cases similar to those out of which they were originally formed, is practical science. The art of medicine then, as it now exists, is the offspring of the science, and as such must look for all its improvement to the bounty of its parent. The time is long gone by when men could regard individual facts in any other light than as suggestions to new enquiries, hints for practice in the absence of distinct rules, and solitary materials for the formation of theories.

Keeping steadily in view the distinction thus laid down between the science and the art of medicine, and regarding the science as the true and only source of the art, let us confine ourselves at present to an examination of the science. Here we encounter the necessity for a more precise definition of the term *science of medicine* than that which we have incidentally given in distinguishing the science from the art. In a certain general sense of the term, the science of medicine may be said to consist of an assemblage of auxiliary sciences bearing either directly or remotely on the knowledge and cure of diseases, of those sciences which the student of medicine is required to know before he can commence the practice of his profession. The following scheme presents these sciences at one view, and enables us to explain the meaning which we propose to attach to the term science of medicine.

1. Anatomy (descriptive and structural), comparative anatomy, morbid anatomy; or the sciences of healthy and diseased structure.

2 Botany (with certain other parts of natural history), chemistry, materia medica; or the sciences descriptive of the materials used in the cure of disease, including the modes of preparing, preserving, and compounding them.

3. Physiology, pathology; or the sciences of healthy and diseased functions.

4. Therapeutics; or the science of healing, including all the knowledge which we acquire by experience or experiment of the virtues of remedies.

5. Medicine, surgery, midwifery, forensic medicine, hygiene; or the practical applications of the knowledge furnished by the foregoing branches of science.

The first two groups consist of sciences of description which owe their origin and improvement to the faithful exercise of the senses on visible and tangible objects, and rarely require the use of induction, or the extensive collection of similar instances which must precede the application of that powerful instrument of discovery. Morbid anatomy, indeed, at first sight appears to form an exception to this statement, but it is only when it is studied in immediate connexion with diseased function. The third and fourth groups (including physiology, pathology, and therapeutics,) bear directly on the investigation and cure of disease, a knowledge of healthy function being an essential preliminary to an investigation of disease, an acquaintance with disordered function being equally essential to its identification, and a knowledge of the virtues of remedies to its cure, whilst morbid anatomy studied in immediate connexion with pathology, instructs us as to its cause. These four sciences then, physiology, pathology, therapeutics, and morbid anatomy, brought to bear on the study and cure of diseases, constitute what we have chosen to term the science of medicine. This definition is adopted, not for its accuracy, but for its convenience, as it brings together all those sciences which stand most in need of improved methods of investigation, and thus serves to make our enquiry more definite. Surgery and midwifery are not excluded from this definition, at least only such parts of them as are merely mechanical; and forensic medicine and hygiene, in as far as they require similar methods of investigation, may benefit indirectly by our enquiries.

We now return to the assertion with which we set out, that medicine considered as a science is most imperfect. Those who are about to enter upon its studies should know this; those who have completed them need scarcely to be reminded of a fact which every day's experience must impress more and more strongly on their minds. Now to what cause or combination of causes are we to attribute the acknowledged imperfection of medicine as a science, and its consequent difficulty as an art? Is that imperfection necessary or accidental? In other words, is it inherent in the subject itself, or does it depend upon want of sufficient materials, want of industry in its cultivators, or on some defect in our methods of investigation? These questions are of sufficient importance to merit a detailed examination. The only way to answer them satisfactorily is to compare medicine with other branches of human knowledge, and especially with those which have attained to the greatest degree of perfection.

There are certain "primary existences and relations," to use the words of Herschel, "which we cannot even conceive not to be, such as space, time, number, order, &c." Not only can we not conceive them not to exist, but all men who think about them must have very nearly the same notion concerning them, though they might find some difficulty in giving a satisfactory definition of them. Now the measure of space and time and indeed of quantity and magnitude in general, is number, and number has the same meaning for all mankind, and is the only thing about which difference of opinion is impossible. As, moreover, numbers are altogether independent of the things counted, and admit of being represented by symbols which are free from the errors of ordinary language, it is obvious that we can reason upon numbers as we can reason upon nothing else. The science of arithmetic, and of algebra (which is merely a more general and powerful arithmetic) being free from the errors of sense on the one hand, and the errors of language on the other, is as far as it goes a perfect science. Geometry too, and the whole circle of what are called the pure mathematics, being sciences of quantity, and therefore, in a certain sense, of number, partake of the absolute certainty of the sciences of arithmetic and algebra. Arithmetic and algebra deal with number without reference to the nature of the things counted, and they are busied in preparing instruments of calculation, as delicate as powerful, for the service of all science. The geometrical sciences resemble arithmetic and algebra in treating of relations of matter not less simple than that of number, such as magnitude, distance, and relative position, carefully excluding all reference to the material of which the things measured consist, and applying at every step the instruments of calculation furnished by the sciences of number. By this union of the numerical and geometrical sciences, fresh instruments of calculation are prepared ready to be applied to a thousand purposes, whether scientific or practical.

The first and simplest use to which these instruments of calculation were put was in the science of astronomy. This science is *par excellence* a science of numerical relations. Magnitude, distance, position, motion, and time are its elements, all of which admit of being expressed by numbers. Its facts are the simplest observations made by the eye, the most certain of our senses, aided by the telescope, the most complete instrument of man's invention, and by other mechanical contrivances remarkable for their accuracy no less than for their ingenuity. Matter enters no otherwise into the calculations of the astronomer than as it has bulk or distance, or relative position. His observations made, he follows out his calculations at his leisure in his study, representing his suns by points, and his distances by lines, sketching out his schemes of an universe on paper, following the track of a planet with his pen, foretelling future events as if he had himself planned them, and awaiting with tranquil confidence the fulfilment of his predictions. Calculation is the secret of the perfection of his science; it is this which gives it all its certainty; it is this which places it at the head of all other sciences, and makes it the model of a perfection which all of them strive to imitate. Here then we have the example of a science of *observation* scarcely inferior in certainty to the pure mathematics themselves, the work of unassisted reason.

But there are other properties of matter almost as simple as those which we have already specified, which admit of the application of the same instruments of calculation, and form the materials of sciences scarcely less perfect than astronomy itself. Such are weight, or to speak more correctly gravitation, and motion, of which the measures are distance and time. The application of calculation to these properties of matter constitutes the sciences of statics and dynamics, hydrostatics, and hydrodynamics—of matter in its two states of solid and liquid, at rest and in motion. In the case of dynamics, force is measured by motion, and motion by the distance traversed by the moving body in a given time. Now the distance may be represented by a straight line, and the time by a symbol, and the direction of the motion by an angle, so that here, as in the case of astronomy, the results of observation or experiment may be worked out in the closet, expressed in figures or traced with the pen. As the amount and effect of a single force may be represented by a straight line, so may the results of two forces acting on the same body be presented to the eye by curves. It is thus that the philosopher with his pen in his hand, and shut up in his closet, reasons upon lines and angles and triangles, and works with symbols and figures, until he succeeds in rearing sciences as true as they are practical, embracing with equal ease the smallest and the grandest objects, now tracing out the motion of an atom, and now following the planets in their course.

Optics, another science in which calculation plays an important part, has, like those just mentioned, attained a high degree of perfection. Here, too, the rays of light admit of being represented by lines, and their direction by angles; and symbols and numbers are employed as measures of quantity. But not to multiply examples, (for our space will not allow us to extend this introductory part of our subject), suffice it to observe that all sciences which deal with those simple relations and properties of matter which admit of being represented by lines or figures, or symbols, and are in their very nature numerical, may attain to very high degrees of perfection.

It will be observed that all the sciences of which mention has been made make choice of one or two simple relations and properties of matter, and apply to them the subtle and powerful instruments of calculation already prepared by abstract reasoning; and that the observations or experiments which form the staple of those sciences are of the very simplest kind. Their certainty and perfection bear an exact proportion to the accuracy of the observations or experiments which they employ, and the extent to which they avail themselves of calculation.

It must not be supposed that even in the sciences of which mention has just been made all is certainty, and that no fallacies of any sort exist. Out of the pale of the pure mathematics there is no absolute certainty; the most perfect of the mixed mathematical sciences has its probabilities. Thus, to take astronomy as an example, wherever the senses are employed there may be fallacy, wherever instruments are used there must be error, and though it may appear a task of no great difficulty to ascertain the precise position of an object, or the relative position of two objects with regard to each other, experience shows that this cannot be effected with perfect precision. How, then, does the astronomer rectify the errors of his observations? Why, by multiplying them. For instance, he wishes to determine the precise position of two stars, but his instruments are not perfect nor his senses sufficiently delicate; and he finds that one observation makes the distance between the two objects too great, and another makes it too small, and a third and a fourth differ from both of them and from each other; and he may make hundreds of observations without finding any two precisely alike. What then does he do? He has recourse to the NUMERICAL METHOD—to that method about which medical men are still doubting and arguing, as if it could admit of any question, whether it ought or ought not to be applied. The astronomer takes the mean of all the observations which he has been able to procure, and these often amount to many hundreds or thousands, and adopts that mean as the nearest possible approach to the true number required. Here then the medical man has the example of a science remarkable for the perfection which it has attained, owing that perfection mainly to the use of instruments of calculation of absolute certainty and infinite power; but nevertheless employing the very method which he in his ignorance and inexperience is tempted to reject. Ought not this fact alone to remove his prejudices? Ought not the high authority of the most perfect science existing to weigh with him more strongly than a thousand arguments? It was by this very method on which the medical man places so slight a value that Laplace made some of his greatest discoveries.

Descending from the high ground occupied by these favoured sciences, and passing hastily by those of heat and electricity, which admit of the application of numbers, but await a more complete development, we encounter sciences which still make extensive use of calculation, but own a closer dependence on the exercise of the senses. Amongst these chemistry holds a prominent place, and deserves the attntion of the medical man as forming an essential part of his education. The properties of matter which are examined by the chemist are much more numerous than those which engage the attention of the astronomer or the student of the statical and dynamical sciences. Availing himself of the labours of the geologist, mineralogist, botanist, and physiologist, in bringing together the materials on which he is to operate, he submits to a minute and careful analysis all the material substances which the earth affords, resolves them into their simplest elements, and tests where possible the accuracy of his analysis by the success of his synthesis. In the prosecution of his task he receives powerful assistance from agents which he has learned to create at will: fire and the several electric fluids become his ready instruments in subduing the stubborn matter from which they themselves were generated, and one form of matter aids him in bringing another under his control. Furnished in this manner with powerful assistance in the prosecution of his enquiries, he provides himself with instruments wherewith to measure the intensity of the several influences by which he surrounds himself. The barometer, the thermometer, the pyrometer, the hygrometer, and the balance give minute accuracy to all his results, and express those results in numbers. Figures, indeed, pervade the whole science of chemistry; all its statements wear a numerical form, and its theory of definite proportions, like the astronomer's theory of gravitation, is a numerical theory. It was this numerical theory which converted the art of chemistry into a science; it is this which lends it its chief attraction as a study; it is this which gives precision to all its practical operations.

In the case of chemistry, as in that of astronomy, the instruments which are employed are not, and cannot be, perfect; and sources of error may mingle with the most careful analysis; hence, as the astronomer multiplies his observations, so does the chemist his experiments, and both alike adopt the numerical method as a means of ensuring the accuracy which they prize so highly. The chemist owes all the power which he gains over matter, and all the knowledge which he accumulates concerning it, to his entire command of the things upon which, and the instruments with which he operates: the one are passive in his hands, the other obedient to his will. The perfection of his science springs from calculation, the certainty of his art from the power which he has of making the matter he employs identical with that of which he has already ascertained the properties. The element which he has once procured in its purity by his analysis, he can use in the same purity in his synthesis. The chemist triumphs over unorganized matter, decomposing and recomposing it at will, but organized matter still baffles all his attempts to reconstruct it, however perfectly he may have succeeded in resolving it into its original elements.* Here then we get the first glimpse of an essential distinction between the world of unorganized and the world of organized matter.

Chemistry investigates one order of properties only with which matter is endowed—its atomic affinities; but these are by no means the only ones which man is interested in understanding. The mechanical properties and uses of matter are in some respects still more important. The instruments which he makes use of in his scientific enquiries, and the materials which he employs in the arts of life are wrought out of the va-

* Some few of the excretions form an exception to this statement.

rious forms of matter which surround him, and he is deeply interested in gaining an accurate knowledge of their mechanical properties. This knowledge can be obtained only by repeated experiment. If every form of matter which goes by the same name had precisely the same properties, and could be identified without difficulty, one careful experiment would suffice to teach us all we want to know concerning it. But this is far from being the case. The rough materials which man finds ready to his hand, and those of which he is in a certain sense the maker, resemble each other without being absolutely identical. To ascertain the mechanical properties of the wood, or stone, or iron, which he uses in his buildings or his machines, he must make not one but many experiments, and must take the mean of all as the nearest possible approximation to the true value of which he is in search. So that here again we have the application of that numerical method, which we have already seen doing good service in the hands of the astronomer and the chemist.

All that we know then of those sciences which have attained the highest degrees of theoretical perfection and practical exactness convinces us that they owe these great advantages to calculation. All scientific experience points to this as the grand secret of their superiority, the source both of their certainty and their power. Calculation, as has been well observed, is the very "soul of science," the universal measure of all our knowledge, the sure guide to all its practical applications. Astronomy and chemistry, the one a science of observation, the other of experiment, owe the perfection they have reached mainly to this cause. A numerical theory embraces and combines all their scattered facts, and presides over all their operations; the instruments which they employ give their indications in the language of numbers; and the errors to which they are liable are corrected by a numerical method. But of these two sciences there can be no doubt which is the most perfect. Astronomy holds the first place, because it has to do with simpler relations of matter, employs fewer instruments in its service, depends less upon the uncertain exercise of the senses, and possesses a more comprehensive theory. All that has now been stated, then, leads to the conclusion that the certainty of a science is mainly determined by the extent to which it admits of the application of numbers.

The foregoing observations apply merely to those sciences which have to do with unorganized matter, and its relations and properties; and though they point to the principal source of their perfection and power, they do not reveal the whole secret of their superiority. Taking the practical applications of science as the test of its perfection,—and we know no other or better test,—we at once discover a sufficient cause of the acknowledged superiority of the sciences which deal with unorganized matter over those which have living beings for their study and object. Astronomy, as has been already stated, is more perfect than chemistry, because it deals not with *properties*, but with *relations* of matter. Among those sciences which deal with properties of matter, that must needs be most perfect which has to do with matter which either is, or may be made to be, identical with all other matter bearing the same name. This is the peculiar privilege of the chemist. He can procure several forms of matter in a state of complete purity, and having once examined all their properties, and given to each an appropriate name, he can reproduce them at will, combine them at pleasure, and foretell the results which will take place with perfect ease and certainty. Not so, however, with the mechanic or engineer. He uses materials which, bearing the same name, resemble each other without being positively identical; the several fragments of wood, and stone, and iron, which he employs in his fabrics, are not in every respect the same, though they pass by the same name, and are sawn from the same tree, or hewn from the same quarry, or smelted in the same furnace. If availing himself of experiments on the strength of any one specimen of these materials, or even of an average of several trials, he were to construct his fabrics of merely that degree of strength which such previous trials indicated, he would often find his best labours thrown away. To ensure solidity and durability to the works of his hands, he must remember that his materials are not identical with those which bear the same name, and he must be prepared greatly to surpass the limits of the possible variation in their properties.

We have now the date necessary for the solution of our first question, *Are the imperfections of medicine considered as a science inherent in the subject itself?* They are. The physician, unlike the mathematician, is not the creator of his own science; unlike the astronomer, he has no simple relations of matter to deal with; he cannot, like the chemist, make any two things which he examines or uses identical; the objects of his study are more variable than the winds and tides, and the materials with which he works infinitely more difficult to adapt to their uses than the matter which the mechanic or the engineer presses into his service. In all his preliminary studies (with the exception of inorganic chemistry), in all his original enquiries, in all his practical applications, he encounters the varying effects and complicated phenomena of Life. The human frame unites within itself all that is most wonderful in contrivance and most elaborate in workmanship. Its structure as much surpasses the most skilful work of man's hands, as its functions do the play of his most ingenious mechanism, and its products the results of his most refined chemistry. That which he knows bears no proportion to that of which he is entirely ignorant; what he sees he sees but darkly; much of what he does he does but guessingly. He seeks for causes, but they elude his search; the vital principle which contains the solution of his difficulties baffles him at every turn; he strives, as it were, to seize it by force, but the violence which he uses defeats itself, and the tortured body dies that it may preserve the secret of its life. Such, and so inscrutable is the body in health; disease surrounds it with new mysteries. Its structure passes through strange transformations, its functions undergo wonderful changes, a new chemistry presides over its secretions, and new principles seem to pervade its every part. Exposed from without to a thousand varying influences; subject within to innumerable changes; governed by a subtle principle which pervades every part, but seems to have no single centre of action; the tenement and instrument of a mind which it both obeys and governs; the human body forms, beyond all comparison, the most difficult, the most complicated study which offers itself to our choice.

Imperfect our knowledge of such a structure is, and must ever be; if we could confine our enquiries to a single human being, or even if every human being were in all its parts and all its functions, the counterpart of every other; if external influences produced the same effects on all, and internal changes followed the same march in all; even then we could scarcely hope successfully to fathom so many mysteries, and unravel such intricate combinations. But so far from one human body being identical with or even similar to every other, each differs from another, in outward form, in inward structure; in health, in disease; in the degree of influence which external things exert upon it; in the effects of food and remedies. The food which nourishes one man shall act as a poison on another, the remedy which produces one effect in one case shall be powerless in a second, and in a third shall have an effect the direct reverse of that which it usually produces. But passing by these idiosyncrasies, as being of rare occurrence, and therefore comparatively unimportant, we encounter in human beings themselves, and in the several parts and functions of their bodies, differences in degree not less extraordinary than those differences in kind. If, for instance, we compare two persons of the same age and sex, both in the enjoyment of what we term perfect health, we shall find that they differ widely in size, in stature, in strength, in complexion, and in feature; their minds, too, as far as we have the means of judging of them, are as different as their bodies. If from the general appearance we turn to the individual functions performed by the several parts of their frames, we discover differences in degree, if possible, still more extraordinary. Taking as an example the function of the circulation, as that for which we have the most exact measure, the pulse of the one shall beat fifty times in a minute, that of the other nearly twice as often, the respiration shall vary within as wide limits, and differences scarcely less marked shall be discovered in all the other functions. Suppose the same parties seized with the same disease, can we doubt that they would exhibit differences as strongly marked in their symptoms; that they might require corresponding differences in treatment; that the same treatment might cure the one which proved fatal to the other; or that if the disease proved fatal in both instances, we should discover some striking structural changes in the one, not present in the other? We should, moreover, find the original structure of the one in every part of the frame different from that of the other.

Such being the intricacies of the framework, and of the functions of the body in health and disease, let us next enquire what means we have of unravelling them. None but the diligent exercise of the senses, and that not ignorantly, but knowing that which has been done by others; not passively, but actively; not carelessly, but with a plan; not aimlessly, but with an object. Time was when men thought to make discoveries by reasoning upon abstractions, and playing with words; imagining that because reason could count and measure, she could almost dispense with the aid of the senses, and find out what nature did by merely speculating upon what she ought to do. Happily for the cause of science, men have been taught the right use of reason, and the true value of observation and experiment. The method which Hippocrates practised and Bacon enforced has become with us a habit. "*Homo, naturæ minister et interpres, tantum facit et intelligit quantum de naturæ ordine re vel mente observaverit, nec amplius scit aut potest.*" These words describe the true relation in which man stands to nature, and trace out the eternal limits of his knowledge and his power. The physician describes the source of his knowledge in five words, "Ars medica tota in observationibus." But the term observation is here used in its true and not in its vulgar meaning; not as the mere passive exercise of the senses, but as the union of thought and perception; of thought electing an object, maturing a plan, guarding against every source of error, inventing instruments, improving methods, arranging and classifying the facts collected, and lastly, submitting them to analysis. The simple employment of the senses is not observation, nor is the frequent exercise of them experience: it is, in the true sense of these terms, that the one is the parent of the science of medicine, and the other of the art.

Observation, then, in this its highest sense,—observation invested with the power of experiment,—is the source of all the knowledge which we have obtained or can hope to obtain of the human body in health or disease. But observation, with a few rare and doubtful exceptions, is the source of all other knowledge. Why then are improved methods of investigation more especially necessary to the physician? Because medicine is, beyond all comparison, more difficult than any other science; because it is, in all its parts, a science of observation; and because the objects which it embraces are infinitely more variable and more complex than any others which man can contemplate. The only science which admits of any comparison with it in this respect is Meteorology; but even this, imperfect as it is, and much as it depends upon observation, does not present a tithe of the complexity of medicine. The most compound meteorological changes are brought about by comparatively few elements, and these individually admit of the application of accurate instruments. The thermometer, the barometer, the hygrometer, the electrometer, and the anemometer, (with the exception of the last, instruments of very accurate construction,) apply a numerical measure to the principal elements of all atmospheric changes, and yield assistance far more powerful than any which the physician can ever hope to obtain. With few exceptions the elements which make up the complex phenomena of health and disease admit of no exact measure, and must be described in the inexact and imperfect language of the senses. When the physician has done all he possibly can with the few instruments which he possesses, he has done little towards an exact description of a disease. He can count the pulse, but its frequency is only one measure of the circulation; he may, in like manner, register the number of respirations; but here again he has but one comparatively unimportant indication of the state of the respiratory function; he may ascertain the temperature of the body, and weigh and test its excretions, but after he has done all this, he has only begun his description of the disease. Then again its causes, its consequences, and the effects of the teatment adopted are to be carefully enquired into, and every day's progress to be recorded with a minuteness which fatigues and disheartens the observer. And yet all these things are to be attended to, and all these things are varying every day and every hour. Compare with this complication, this almost inextricable confusion, what science we will, we shall find none half so difficult as this; none which stands so much in need of every aid which experience can suggest, or ingenuity invent.

We might, without much difficulty, have extended the foregoing remarks, for which we are chiefly indebted to the short essay of Dr. Guy,* so as to include other causes of the imperfection of medical science, and the comparative superiority of certain other branches of human knowledge. As our space will not allow us to extend our enquiries further, we shall content ourselves with throwing into the form of short propositions some of the principal causes of the advantages which the more favoured sciences possess, and of the necessary disadvantages under which medicine is placed.

1. The only sciences which are necessarily certain are the arithmetical and geometrical sciences; of which the object is to furnish "instruments of calculation," of infinite power and absolute certainty for the service of all the other sciences.

2. The extent to which these instruments of calculation can be applied to the objects of sense is the true measure of the perfection of the sciences.

3. The objects of sense are either *relations* or *properties* of matter: and the relations of matter being more simple than its properties, and at the same time more easily submitted to measurement, the sciences which deal with the relations of matter are more perfect than those which have to do with its properties.

4. Of the sciences which deal with *relations* of matter, those are the most perfect which treat of the simplest relations; in like manner, of the sciences which have to do with *properties* of matter, those are the most perfect which treat of the simplest properties.

5. Those sciences are the most perfect which deal with the simplest relations or properties of matter, admit of the most extensive use of calculation, employ the most perfect instruments, and possess the most comprehensive numerical theory.

6. Medicine is necessarily a most imperfect science, because it treats of the most complex properties of matter in its most complicated form, (that is to say, organized;) because it does not admit of the use of the most perfect instruments of calculation; because it possesses very few accurate instruments compared to the great number of objects to be examined; and because it has no numerical theory.

7. Lastly. Medicine considered either as an art or a science is and must continue to be imperfect, because the several objects of its study, though they bear the same name, neither are nor can be made counterparts of each other.

Medicine, therefore, as compared with other branches of human knowledge, is, and must necessarily be, imperfect; but though this be admitted to the fullest extent, it does not follow that it allows of no improvement. On the contrary, it may be safely affirmed that there is no science existing which admits of so much improvement. If this be true, in what direction, it may be asked, shall we look for the means of its advancement? What are the remediable defects under which it la-

* On the Value of the Numerical Method as applied to Science, but especially to Physiology and Medicine. By William Augustus Guy, M.B., Cantab., Professor of Forensic Medicine, King's College, London. Reprinted from the Journal of the Statistical Society of London. 1839.

bours? what the impediments to its progress which we may reasonably hope to see removed?

We have already stated that the foundation of medical science must be laid in observation, in the sense in which we have employed that term. But the foundation must be much broader and deeper than it is, before we can hope to see a superstructure reared upon it worthy of the name of a science. And yet at first sight there would seem to be no want of facts. Our journals, our reviews, our monographs teem with facts enough to construct a dozen sciences; our press is most prolific in single cases, and small groups of observations, nor is it altogether barren of collections on a larger scale. But these are as nothing compared to the wants of so vast a subject. We not only want many facts, but we want many *comparable* facts. Putting aside our false facts as worse than useless, our imperfect facts as deceptive, our unmeaning facts as impertinent, and our wonderful histories as the mere curiosities of medical literature, how few will remain which can be safely employed in the building up of a science of medicine. What we want then, in the first place, is a greater number of comparable facts. These facts must be formed into groups, these groups must admit of comparison with other groups; that which is common to all the facts must be expressed in language at once concise and intelligible, in language which may, like the single facts themselves, admit of strict comparison. There is no language which answers to this description but the language of numbers, and the language of numbers is the language of science. The object of the foregoing remarks has been to prove this, and to place medicine under the obligation of listening to stronger arguments than those of reason, the arguments of example and authority. We are advocates then of the *numerical method*, not as a method adapted to the use of this or that science, but as a universal method indispensably necessary to the advancement and improvement of all the sciences of observation and experiment.

The term numerical method is here used in preference to the word statistics. Properly speaking, statistics means the science of states, (from the German *staat*,)* and it is nearly synonymous with the terms "political science," "political economy," "social science." The name owes its origin to Achenwal, professor of history in Göttingen, who published an historical work in 1749, in which the term *scientia statistica* occurs for the first time. The use of numbers as a means of comparison in this work of Achenwal led to the strange mistake of regarding their employment as a new method, and after a time every application of numbers in the service of the sciences of observation was dignified with the sounding title of statistics; and we have at last become as familiar with the term medical statistics as with the phrase medical science. We are interested in pointing out this mistaken use of the term statistics because, as we have already stated, we wish to show that so far from the employment of numbers in medical investigations deserving to be characterized by a new word, or looked upon as an innovation, their non-employment till a recent period proves that medicine has profited little by the ex-

* Dufau, in his Traité de Statistique, derives this term from the Latin status.

ample of the more perfect sciences, and gives us good ground for hope that improved methods of investigation may do much to retrieve its character, confer upon it a greater degree of certainty, and enlarge the sphere of its practical usefulness.

The numerical method is sometimes erroneously regarded as a mere substitution of figures for words. Against this mistake Gavarret strongly protests, and with good reason, though the mere substitution of figures for words is a great improvement in our scientific methods, seeing that figures admit of strict comparison which words do not. "The *sometimes* of the cautious is the *often* of the sanguine, the *always* of the empiric, and the *never* of the sceptic; but the numbers 1, 10, 100, 1000, have but one meaning for all mankind." If this sentence embodied all the advantage to be expected from the substitution of figures for words, it would furnish strong reasons for the exclusive use of figures; but the numerical method, as we have just stated, is something more than this. It is not merely a language, but a science. Just as the pure mathematics furnish the instruments of calculation for the service of those sciences which deal with fixed and certain quantities and measures, so does the numerical method supply instruments of calculation for varying quantities, and for events brought about by many conjoined causes. We have already spoken of its employment by the astronomer, the mechanic, and the chemist, for the purpose of correcting the errors of observation and experiment; we have now to speak of other and higher benefits which it confers on the science of the physician.

The science of medicine is conversant with more than one order of facts. For the most part it has to do with what may be termed compound facts; that is to say, with large groups of circumstances. Such for instance are diseases traced through their entire course, and faithfully described in all their symptoms. But these compound facts admit of being separated into their elements, or into the simple facts of which they consist. Particular symptoms of disease, for instance, may become the subjects of separate consideration and enquiry, or our attention may be confined to the result of the treatment which we employ; in other words, to the event of the disease. Single symptoms, or isolated facts or questions on the one hand, and events on the other, are the proper objects of the numerical method; compound facts require the application of a different method first pointed out by Lord Bacon, and subsequently matured into a uniform plan by the late Dr. Todd, of Brighton.* The first order of facts are the subjects of our present enquiry; the second will be examined on a future occasion.

Symptoms of diseases, or particular facts connected with them, may be studied either as events of more or less frequent occurrence, or as circumstances varying in intensity in different cases, the object being to determine the average degree of that intensity. Considered merely as events, without regard to the degree in which they are developed, the symptoms of disease resemble other events, and the same rules which apply to events in general apply to these in particular. Regarded in this light, therefore, the individual symptoms or circumstances of diseases

* The Book of Analysis, or a new Method of Experience. London, 1831, 8vo.

may be discussed when we come to speak of events in general. But we shall first say a few words on symptoms regarded as of variable intensity.

In order that our knowledge of symptoms in respect of intensity may form a part of the science of medicine it must be complete and accurate; embracing the rule in all its degrees, and the exceptions in all their variety. If the symptom consist in an entire change of some healthy function, that healthy function must be accurately examined, and its healthy state must be our standard of comparison. If, on the other hand, it be something entirely foreign to the healthy state of the body, it must be examined alone, but with equal care. In any case, if it be such as not only to exist in varying degrees, but to admit of being expressed by figures, all the observations which we make with respect to it must wear a numerical shape. For instance, the frequency of the pulse and respiration, the temperature, the quantities of some of the excretions, especially the urine, and the specific gravity of that liquid, admitting of being expressed by figures, ought never to be stated in any other terms, and that our knowledge may be accnrate, it must extend to a great number of cases and embrace every probable degree of intensity and every exception to the rule; and the results must be stated in the same accurate language, the mean as well as the extreme values being in every case expressed. As it is no part of our present object to discuss the practical application of the knowledge thus collected, we shall not attempt to show the use to which both these values may be put in the actual practice of our profession; suffice it to observe that too little attention seems to have been paid to the extreme values in the observations hitherto collected. The number of facts which it may be necessary to collect in any particular instance, in order that we may possess an accurate mean and the real extremes, is not easily determined. The simplest rule appears to be "to divide the whole number of observations into groups of equal size, and compare them the one with the other; if the average value of each group is the same, we may safely conclude that we have arrived at the true mean: if not, we must increase the number of our observations, and the size of our groups, till the desired equality is obtained. For instance, one hundred observations having been collected, are divided into four parts, containing twenty-five observations each; if, on comparing these four parts with one another, we find that they yield the same average result, we have good reason to regard such result as the real average. But if the average results are different, we must divide the one hundred observations into a smaller number of parts, and if necessary, increase the number of our facts. If each collection of fifty or of one hundred observations, as the case may be, yields the same average, we may confidently regard that average as the true one." The same method may be employed for determining the real extremes both in excess and defect.

We now turn to the more interesting and important subject of events brought about by a great number of conjoint causes, all of which may vary within wide limits of intensity in the several instances observed; and where the object is to collect and express numerically the frequency of the events in question.

As an example of such events we will take the alternative of death or recovery, the consequence of a given method of treatment in a given form of disease. Here the causes which combine to bring about the one or the other of these two events are extremely numerous. Gavarret divides them into five distinct groups, as follows :

1. *Individual conditions.* Age, sex, temperament, constitution, previous diseases, and state of health at the time of the invasion of the disease in question.

2. *Hygienic conditions antecedent to the invasion of the disease.* Profession, social position, mode of life, ventilation, state of dwelling, kind of nourishment, moral influences.

3. *Hygienic conditions during the treatment.* Healthiness of the place in which the patient is treated, moral influences which act upon him during the course of his disease, and the exactness with which the orders of the medical attendant are followed.

4. *The disease itself.* Nature of the disease, extent and degree of organic lesions, and of the influence which they exert on the economy, period which elapses between the attack of the disease and the commencement of the treatment, and the several complications which may arise in the course of the disease.

5. *Treatment employed.* This head includes not merely the remedy made use of, but the dose administered, and the various auxiliary remedies used to meet occasional symptoms.

It would perhaps be possible to extend this list on the one hand, and to simplify it on the other; but assuming that it is a tolerably correct representation of the several circumstances which may affect the success of a plan of treatment adopted in any given disease, it must be obvious that, if the disease in question be one of any severity, the event of it may be greatly influenced by any one of these several circumstances, and as it is altogether impossible to estimate the influence which they would severally exert, we must rest contented with an accurate observation of the result obtained in all the instances. Confining our attention, therefore, to the result, what are the rules which are to guide us in obtaining that result, and in applying our knowledge of it to the prediction of the fate of patients labouring under the same malady?

The first thing to be attended to is the quality of our observations, the second is their number. It is obvious that we can scarcely err with regard to the mere event, provided we take the most ordinary degree of pains: in this it will be easy to make all our observations strictly comparable. But there is ample room for error in the selection of our cases, and in the circumstances under which we place our patients during the course of their malady. The disease may be loosely defined, and we may confound together cases of a totally different nature, or we may be calling a mere symptom a disease, and thus throw together under the same title things in themselves essentially different. For example, not long ago, a man would have given himself some credit for an industrious observation of cases of ascites, and would have thought that he exercised discrimination enough if he assured himself that all the cases which he reported were bonâ fide cases of that disease; but his facts would have been almost useless, because they would not have been strictly comparable. What a heterogeneous assortment of observations should we not have had! Ascites from chronic peritonitis, from diseased kidney, from obstruction to the circulation through the venæ portarum, from diseased heart, from certain forms of pulmonary disease, from scarlatina, from cold, &c. all grouped together under the same name, all treated alike, and all leading to one of two results, recovery or death. The observations, then, which we group together under the same name, must be strictly comparable facts, in order that our conclusions may have any value.

But not only must the diseases which we call by the same name be the same things, own the same causes, and have the same things in common, (at least such as are most characteristic, as the albuminous urine in cases of ascites from renal disease,) but the circumstances under which our patients are placed must be, as far as possible, the same. They must occupy the same locality, receive the same attention, partake of the same diet, and, as far as practicable, be placed under the same treatment.

Those things, then, which are in the power of the physician must be made the same in all the cases observed, or the result must needs be faulty. But those circumstances over which the physician has control form but a small part of the influences brought to bear on the several subjects of his observations: there still remains a long list of circumstances peculiar to each individual over which the physician can exercise no control, and these must have a powerful effect on the result of his treatment. The existence of such individual peculiarities precludes the possibility of meeting with any strictly comparable facts, and this circumstance has been unjustly advanced as an objection against the application of numbers to medical enquiries. This objection goes on the mistaken supposition, that numbers being made up of units can have no value, unless the units themselves be absolutely identical. The answer to this objection is at once supplied by the fact already stated, that the astronomer is constantly making use of measurements not strictly comparable, and applying to those measurements the numerical method which we are now advocating. Insurance societies of all kinds are constantly making the most important use of events brought about by causes not less variable and entangled than those of which we now speak, and the results which they attain justify their calculations. By parity of reasoning, then, the physician may expect to derive advantage from grouping together observations not absolutely comparable, and applying to them the same calculations which his experience teaches him lead in the hands of others to true and valuable results. With regard, then, to the quality of our facts, it is sufficient that they be strictly comparable in those particulars over which we can exercise control; or, to use the language of our author, we must take care to secure "*l'invariabilité de l'ensemble des causes possibles.*"

Having provided for the sameness of our observations, as far as it lies in our own power to do so, the next question regards the number of observations which ought to be brought together, in order that the result of our treatment may be a true result. This question brings us to the most important part of the work before us. Our author, as has been already stated, places the objects and use of the numerical method much higher than the mere substitution of figures for words. According to him medical statistics, or, as we prefer to call it, the numerical method, is "la théorie des grands nombres," the application of the calculus of probabilities to the science of the physician, "le complément le plus indispensable de la méthode expérimentale." Adopting the sentiment of Laplace, " Le système tout entier des connaissances humaines se rattache à la théorie des probabilités," he proceeds to apply the calculus of probabilities to the solution of some of the most important questions which can engage the attention of the physician. Amongst them the most important is the result of the treatment which he adopts.

The first principle on which our author insists is, that the number of our observations should be considerable. Now this principle will be readily conceded, for every day's experience convinces us that a small number of observations is altogether insufficient to establish any result whatever on a firm basis, whilst on the other hand it shows the sufficiency of very considerable numbers. Every one knows, for instance, that at games of hazard individuals sometimes gain or lose large sums of money in consequence of what is called a run of good or ill luck. Who that has been in the habit of collecting observations does not remember instances in which the chances of two events being equal or very nearly so, one of them has occurred for many times in succession? Such things are constantly happening to the physician; and not to the physician only, for even the mathematician has occasionally encountered these coincidences. What can more strikingly prove the insufficiency of small numbers than the following instance, drawn from the more exact parts of science. The celebrated mathematician Euler, making use of a certain formula, and giving to the abstract quantities contained in it the values 0, 1, 2, 3, &c. in succession, found that all the resulting numbers up to the 40th were *prime* numbers; that is, numbers which have no divisors, or which cannot be divided into any number of equal integral parts, less than the number of units of which they are composed: hence it might be supposed that the law was general. It happened, however, that in the forty-first term the result was a *composite* number. Can any instance be imagined more conclusive than this as to the necessity for establishing our general principles on a sufficient number of cases. The sufficiency of large numbers of facts, on the other hand, is proved by ample experience. The success of the bank in gambling transactions where the capital is large, and of insurance societies where the tables employed are derived from a sufficient number of facts, are conclusive on this point. The uniform results, too, obtained in successive years from large collections of instances of the same kind is well known. Thus, if we compare the number of male and female baptisms registered in England in 1821 and the nine following years, we find that, in these successive years, for 1000 girls baptized there were 1048, 1047, 1047, 1049, 1046, 1047, 1043, 1043, and 1034 boys.* Taking the first eight of these years (for it seems not improbable that the last may be a misprint for 1043) the difference between the highest and the lowest number is less than 6 in 1000, a difference so small as safely to be disregarded in a general result.

Assuming, then, that the necessity for employing large numbers of comparable facts in forming general conclusions will be readily conceded, the next question which arises is, how many facts will be sufficient to establish such a general result as the efficacy or inefficacy of a given plan of treatment in a given disease? Our readers must not be dis-

* De Morgan on Probability, p. 120.

heartened when they are told, that even the most industrious observers of the present day have fallen very far short of the strict requirements of the numerical method, and the indications afforded by the calculus of probabilities. M. Louis, the justly celebrated medical statistician, falls under our author's censure for having asserted the slight efficacy of bleeding in pneumonia, erysipelas of the face, and cynanche tonsillaris, on the strength of one hundred cases of the first disease, forty-four of the second, and twenty-three of the third, and he lays it down as an undoubted principle that "every *statistique*, in order to furnish admissible indications, ought to consist of *many hundreds of observations.*" If Louis, to whom the profession is under such obligations for taking the lead in this grand improvement lies open to censure, what shall we say of the majority of his followers, and in what terms shall we speak of those who still persist in foregoing the aid of numbers, and in drawing important conclusions from one or two scattered, and not comparable facts.* To bring together such large numbers of observations is, in the present state of medical science, and with the existing opportunities and industry of medical men, impossible: hence it is most important to discover some means by which we may know the degree of reliance to be placed upon smaller numbers of observations, and within what limits the results derived from such small numbers may be looked upon as entitled to confidence.

After what has been already stated, the insufficiency of small numbers of observation will not be doubted, nor will it be difficult to understand how the results of observation will become more and more worthy of confidence as the number of observations increases, until at length the general principles established by their aid may be as firmly relied on as the most certain truths of the pure mathematics. Few, for instance, will be inclined to withhold their assent from the proposition established by the facts already quoted, that, in this country, the number of male births is to the number of female births as nearly 21 to 20, and they will scarcely consider the evidence on which this fact rests as less satisfactory than the evidence of their own senses and reason that two straight lines cannot inclose a space. But the question still recurs—are there any means by which we can ascertain the degree of reliance to be placed upon any given number of observations, or by which we can determine the limits of error to which they are subject? THE CALCULUS OF PROBABILITIES supplies us with a method, a method which we may employ with confidence for precisely the same reason that we may use numbers with confidence, namely, the success which has attended its use in the hands of the cultivators of the more certain sciences. To develope this method, and to give examples of its application, is the chief object of Gavarret's excellent work, which we strongly recommend to the perusal of all who are interested in the advancement of the *science* of medicine.

It is necessary to take this method for granted, as it can only be understood by those well versed in mathematics, and it must be received on

* It must be borne in mind, that Gavarret's observations refer to events brought about by vast numbers of external and internal causes combined, and not to states of system which depend upon internal causes alone, such as the functions of the body in health and disease. In this case, though our observations must be often repeated, they need not be so numerous.

the same authority on which we receive the other methods employed by the astronomer, namely, the perfection of his science and the success of his predictions. It is asserted, then, on this high authority, that where an event has been *observed* to happen a certain number of times in a given number of cases, the probability of its happening is not represented by the actual number observed, but lies between limits somewhat greater and somewhat less than that number; and that these limits vary with the number of observations, being wider as the observations are few in number, and approaching each other more and more as they increase. The following example, taken from tables furnished by Gavarret, will sufficiently explain our meaning.

Supposing two collections of observations on the efficacy of a certain remedy in a certain disease to be made, one consisting of 300, the other of 1000, and that the number of cures effected is in each case nine tenths of the whole number of cases; that is to say, suppose 270 to recover out of the 300 cases, and 900 out of 1000; then the numbers 270 and 900 do not give the same degree of confidence, but are subject to differing degrees of error, and are only approximations to the truth. The cures in each instance amount to nine tenths of the whole number treated, or to ·90000 in ·100000. Now for the smaller number 300, the limits of possible error will be ·048990 added to ·900000, or ·948990; and ·048990 taken from ·900000, or ·851010; whilst for the larger number 1000, the limits will be ·026833 added to ·900000, or ·926833, and ·026833 taken from ·900000 or ·873167. In other words, when 300 observations only are collected, the results obtained are much wider from the limits which calculation assigns than when 1000 observations are employed. Supposing for instance that a person having observed 300 cases of any disease, states that 270 cures were effected by a certain remedy, and concludes from this fact that 9 out of 10 patients will be cured in future by the same means, we start forward with the objection—"Your facts are not sufficiently numerous to warrant such a statement; it may have been so in your 300 cases, but 300 observations of any sort whatsoever are liable to a certain fallacy, which can be measured by calculation; and on applying this measure we find that you are not justified in foretelling 9 recoveries in 10 in future, but merely some number between 95 and 85 per cent. Even if you had collected 1000 cases and found that $\frac{9}{10}$ of your cases recovered, all that you could assert for the future would be, that some number between 87 and 93 per cent. would get well."

In the Appendix to Gavarret's work some interesting examples are given, illustrative of the insufficiency of small numbers of facts. We will select one only of these.

M. Louis, in his "Recherches sur la Fièvre Typhoïde," has endeavoured to illustrate the treatment of this disease by carefully analysing 140 cases. The results of these cases are as follows:

52 deaths; 88 recoveries: Total 140.

The mortality therefore is $\frac{52}{140}$, or 0·37143. If we take this result as a strict expression of the efficacy of the treatment adopted in this disease, we shall have the following proposition. Under the influence of the treatment adopted the mortality of typhus fever is represented by 37,143 deaths in 100,000 cases, or approximatively 37 deaths in 100 cases.

If now we make use of the calculations already referred to, we shall find that this assertion is subject to considerable correction, and that the mortality, instead of being exactly represented by the results of this small number of cases, may vary within the following limits:

$$0{\cdot}37143 + 0{\cdot}11550 = 0{\cdot}48693, \text{ and}$$
$$0{\cdot}37143 - 0{\cdot}11550 = 0{\cdot}25593.$$

Thus all that we learn from this limited experience is this—that under the treatment adopted the mortality may lie somewhere between 48,693 and 25,593 deaths in 100,000 cases, or approximatively between 49 and 26 per cent. In other words, that on employing precisely the same treatment upon a very large number of patients attacked with typhus fever, we may lose from a quarter to a half of our patients.

For further illustrations of the necessity for the employment of the calculus of probabilities as a corrective of the results deduced from small numbers of facts, we must refer our readers to the original work.*

The calculations of which we now speak have nothing whatever to do with the *nature* of the facts observed, but merely with their number, and hence any objection which may seem to lie against the employment for medical purposes of formulæ supplied by the pure mathematics, will at once disappear. The only authority which need be adduced in support of this application of numbers to observation is that of the successful cultivators of the most perfect sciences, who employ the same method in their accurate investigations, just as they employed the numerical method long before it was used by the medical man.

The questions which we propounded are now answered. The imperfection of medicine as a science is partly due to the inherent difficulty of the subject, and partly to defective methods of investigation. We have not enough facts, and our men of science are not industrious enough to supply the large demands which are made upon them.

The object of the foregoing remarks is to make the more perfect sciences an example to our own. By showing how important a part calculation plays in those sciences which have attained the highest degree of perfection, we think that we have pointed out the true path to the improvement of the science of medicine. The substitution of figures for words, though a step gained, is not all that is required: the numbers which we deal with must be large ones, and where they are not so, we

* For the benefit of such of our medical brethren as are conversant with the mathematics, we subjoin the formula by means of which the limits of error are obtained.

If $m$ represent the number of times that one of two events (call it A) has happened, $n$ the number of times that another event B has happened, and $\mu$ the total number of observations collected, so that $m + n = \mu$; then the number which expresses the observed frequency of the event A, is not the true number, but merely an approximation to it more or less close as the number of observations is greater or less. That number will in any case lie between

$$\frac{m}{\mu} + 2\sqrt{\frac{2 . m . n}{\mu^3}}$$

and

$$\frac{m}{\mu} - 2\sqrt{\frac{2 . m . n}{\mu^3}}$$

or at least, there are 212 chances to one in favour of its being comprised within those limits.

must make allowances for error proportioned to the number of our observations. To make the principles of our science as close approximations to truth as possible, the facts from which those principles are deduced must be very numerous. Where objects are to be examined of variable magnitude, we must make as many observations as shall include all the varieties which nature presents; where events are the subjects of our enquiry we must collect as many instances as shall enable us to find at least very close approximations to the actual frequency of their occurrence. But the units of which our numbers consist must be comparable facts. Where to obtain these facts is the difficulty. How few men possess the opportunity, how very few the inclination to make such large collections of facts! and what reward can they expect for their industry? The *practical* man who delights in single cases, and glories in those who deal with them, will laugh to scorn the plodding collector of facts by the hundred, and confound the principles which he draws from them with the hypotheses of some dreaming enthusiast. He who devotes himself to the science of medicine must expect little sympathy from the mere votary of the art. And though the science is, and must ever be, the parent of the art, the man of science must look for little gratitude from those on whose behalf he labours—the mere practitioners of the art. Where then must he look for his reward, whether of fame or wealth? Something of the one he may gain from posterity, but little of the other from his contemporaries. He must love science then for its own sake, and labour for its advancement without hope of recompense, satisfied if he leaves the most imperfect science of the day somewhat more certain than he found it. In the meantime he must be prepared to sacrifice those advantages on which men set the greatest store, and for which they make the greatest exertions. We might add much more on this topic, but we are warned to bring our remarks to a close.

Once more, then, the SCIENCE OF MEDICINE wants facts—comparable facts—numerous facts: well observed, carefully arranged, minutely classified, and acutely analyzed. Her language must be the language of figures; her test, the calculus of probabilities; her example, the most perfect and exact among the sciences of observation and experiment.

## Figures and Tables

**Figure f1:**
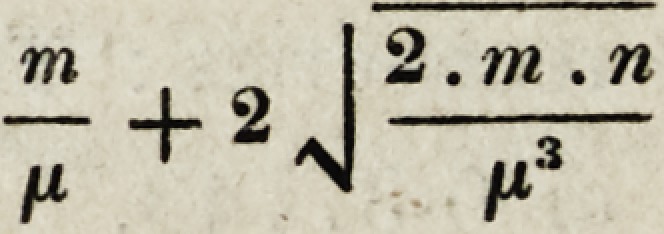


**Figure f2:**